# Cancer incidence and mortality projections in the UK until 2035

**DOI:** 10.1038/bjc.2016.304

**Published:** 2016-10-11

**Authors:** C R Smittenaar, K A Petersen, K Stewart, N Moitt

**Affiliations:** 1 Analysis & Evaluation, Cancer Research UK, The Angel Building, 407 St John Street, London EC1V 4AD, UK

**Keywords:** incidence, mortality, projections, UK

## Abstract

**Background:**

Cancer incidence and mortality projections are important for understanding the evolving landscape for cancer risk factors as well as anticipating future burden on the health service.

**Methods:**

We used an age–period–cohort model with natural cubic splines to estimate cancer cases and deaths from 2015 to 2035 based on 1979–2014 UK data. This was converted to rates using ONS population projections. Modified data sets were generated for breast and prostate cancers.

**Results:**

Cancer incidence rates are projected to decrease by 0.03% in males and increase by 0.11% in females yearly between 2015 and 2035; thyroid, liver, oral and kidney cancer are among the fastest accelerating cancers. 243 690 female and 270 261 male cancer cases are projected for 2035. Breast and prostate cancers are projected to be the most common cancers among females and males, respectively in 2035. Most cancers' mortality rate is decreasing; there are notable increases for liver, oral and anal cancer. For 2035, there are 95 961 female deaths projected and 116 585 male deaths projected.

**Conclusions:**

These findings stress the need to continue efforts to address cancer risk factors. Furthermore, the increased burden of the number of cancer cases and deaths as a result of the growing and ageing population should be taken into consideration by healthcare planners.

Incidence and mortality measures are an important part of cancer control monitoring. These two measures can be further characterised in terms of rates and cases. Age-standardised rates describe cancer incidence and mortality with reference to a standard population making this a measure which is invariant to the size and age composition of the population. Incidence rates can act as a crude proxy for shifting patterns of the prevalence of risk factors linked to the disease within a population. Overdiagnosis can also contribute to increased cancer incidence (Welch and Black, 2010).

Mortality rates are influenced by incidence rates, and also how successful the healthcare system is in diagnosing and treating the cancer under study. However, the relationship between mortality and incidence rates is complex; not all cancer patients will die from their cancer, and survival is improving over time for the majority of cancers. For those that do die from their cancer, there is a time lag between the diagnosis and death, which for many could be several years. Relative survival provides a more accurate measurement of how effective a healthcare system is in diagnosing and treating diseases, as it accounts for the background mortality in the population under study (Ellis *et al*, 2014). However, survival measures are prone to ‘lead time bias', whereby the increased intensity of screening and early diagnosis activities results in many more cancers being diagnosed at an earlier stage (and so potentially extending survival time for cancers without impacting the outcome of the disease; Duffy *et al*, 2008). Screening and awareness measures can also lead to overdiagnosis of some cancers (Welch and Black, 2010), which will artificially improve survival estimates. Understanding changes in incidence and mortality rates is therefore important to public health scientists, as this provides a means with which to evaluate public health interventions. When the risk factors for the development of certain cancers are poorly understood, projections may be the only information available regarding anticipated future burden of the disease.

The number of cancer cases or deaths is the total number of people within a population who have either been diagnosed with or die from cancer, and this is greatly influenced by the size and age composition of the population. This information is critical to understanding and planning for the disease burden.

Here, we used an age–period–cohort (APC) model on current cancer incidence and mortality data for 26 cancer sites and an ‘other' cancer category to extrapolate future trends until 2035. In contrast to predictions, projections do not explicitly include assumptions about changes in risk factors or screening activity for incidence projections, or improvements to treatment for mortality projections (Mistry *et al*, 2011). Although there is a strong link between smoking and lung cancer (Parkin *et al*, 2011), for the majority of cancers the relationship between a single or combination of risk factors is insufficiently strong to be modelled directly (Bray and Møller, 2006). Analogously, improvements in treatments are not modelled directly as they tend to have incremental effects on the mortality rates as opposed to more radical changes, which we would anticipate were a cure to be found. By taking account of age, calendar period and birth cohort, the APC models are able to incorporate historical changes in these components (for example, different risk factor prevalence among different birth cohorts) to make longer term projections (Møller *et al*, 2003; Sedjo *et al*, 2007; Olsen *et al*, 2008). This paper builds on incidence projections for the United Kingdom (UK) presented by Mistry *et al* (2011) by updating with an additional 7 years of data. We additionally present mortality projections. Furthermore, we provide information on case ascertainment from 2000 onwards, sensitivity analyses exploring the impact of several model parameters, projection intervals, as well as a comparison between projections using two standardised populations.

## Materials and methods

We used data on the incidence and mortality of 26 cancer sites and for each sex an ‘other' cancer category (see Supplementary Material A). The ‘other' cancer category is likely to contain a number of different trends of the various cancers it contains which makes modelling more error-prone. However, we have included this category in our analysis as it contains a large proportion of cancer cases and deaths contributing to the ‘all cancers' number. To exclude it would result in an under-estimation of these numbers. The cancer incidence data used for England from 1979 to 2014 were supplied by the Office for National Statistics (ONS) who received the registration data collected by the National Cancer Registration and Analysis Service (NCRAS). The incidence data from 1979 to 2014 for Wales were provided by the Welsh Cancer Intelligence Surveillance Unit (WCISU), and for Scotland by the Information Services Division (ISD) Scotland cancer information programme. The Northern Ireland incidence data are for 1993–2014 and were provided by the Northern Ireland Cancer Registry (NICR). Earlier incidence data for Northern Ireland are not reliable as the NICR was established in 1993. For data between 1979 and 1992, we scaled Great Britain (GB) data up to the level of the UK, by calculating the proportion of the UK population which GB constituted each year by sex and 5 year age band, and used this to scale up the GB incidence to UK level.

The cancer mortality data for England and Wales between 1979 and 2014 were provided (and collected) by the ONS. For Scotland, the data were provided by the Scottish Cancer Registry and collected by the General Register Office (GRO) for Scotland. Cancer mortality data for Northern Ireland were obtained from the NICR (and collected by the Northern Ireland Statistics and Research Agency (NISRA)). Mesothelioma mortality data are an exception. They were provided by the Health and Safety Executive for Great Britain between 1979 and 2014. We used the above scaling method to scale this GB level data up to the level of the UK for 1979–2014.

Incidence and mortality data were split into the number of cases by 5 year age group and sex. Population estimates and projections for GB and the UK by 5 year age groups were obtained from the ONS Population Services. All modelling was completed by 5 year age groups. The age groups were as follows; 15–19, 20–24, 25–29, 30–34, 35–39, 40–44, 45–49, 50–54, 55–59, 60–64, 65–69, 70–74, 75–79, 80–84, 85–89, 90+. We did not model 0–4, 5–9 or 10–14 age groups. There are so few cancer cases and deaths in these age groups that including them would have made the data sparser and therefore had a negative impact on model fitting. Our observed and projected age-standardised rates (ASRs) are for those aged 15–90+, and so will be higher than ASRs for those aged 0–90+. This is also due to the relatively low amount of cases and deaths in the 0–14 age group; by removing these age groups where the risk of being diagnosed with or dying from cancer is very low, rates for the 15–90+ age group are higher as the population the rates are based on has an at least slightly elevated risk of being diagnosed with or dying from cancer in comparison with the 0–14 age groups. Therefore the ASRs in this paper are not directly comparable to ASRs calculated for people aged 0–90+. Weights from the European Standard Population 2013 (ESP 2013) were used to age-standardise these rates. The oldest age group in the cancer incidence and mortality data is 90+, whereas the ESP 2013 has categories for 90–94 and 95+, and therefore we summed the weights of these categories for the 90+ age group.

We used an APC model to model incidence and mortality for each cancer, and then this was extrapolated out to 2035. The basic form of the APC model is:


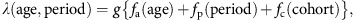


in which *λ* corresponds to the incidence or mortality rate as a function of age and calendar period, *g* is a ‘link' function (either the ‘power 5' function, *g*(*x*)=*x*^5^ (Møller *et al*, 2002) or a log link function), and functions of age (*f*_a_), period (*f*_p_) in terms of year of incidence, and cohort (*f*_c_) in terms of year of birth. The functions *f*_a_, *f*_p_ and *f*_c_ are natural cubic splines. Natural cubic splines are favoured over step functions, because natural cubic splines are flexible, and reflect smooth changes over time, which allow a more biologically plausible way of modelling non-communicable disease data. The APC model contains the date of birth and age of diagnosis (tabulated by 5 year groups), which sum to give the date of diagnosis (i.e., there is a linear dependence between age, period and cohort). Therefore, the model suffers from the identifiability problem. To address this issue, cubic splines were used to absorb the linear trends in period and cohort effects into a drift component. A linear extrapolation was then used beyond the final knot in the spline to project this drift component into the future, with an attenuation applied to this, based on the assumption that these historical trends will not continue indefinitely (Møller *et al*, 2002; Mistry *et al*, 2011; Sasieni, 2012).

We completed sensitivity analyses to determine which combination of either a log or power 5 link function, the number of knots in each of the cubic splines and the attenuation of the drift component was best able to project data over the period of 1999–2014 using a data set that was truncated at 1998. We assumed that the combination of model parameters which provided the most accurate projections using historical data are the most appropriate model parameters for projecting using the current data set (see Supplementary Materials B, B.1 and B.2 for further details). For the incidence data, we used a log link function, with seven, five and three knots in the age, period and cohort splines, respectively, and with a 10% year-on-year attenuation on the drift component. For the mortality data, we used a log link function, with six, five and three knots in the age, period and cohort splines, respectively, and with a 6% year-on-year attenuation on the drift component.

The APC model is available as a function to download in STATA 13 (Mistry *et al*, 2011). All other code for this analysis was developed in-house in STATA 13.

In line with Mistry *et al*, 2011, our methodology takes account of changes relating to screening for breast and prostate cancer. We generated modified data sets to estimate the underlying incidence trends in these cancers before screening, as well as estimated the increases attributable to screening. We used data from the period 1979–1991 – before the introduction of prostate-specific antigen (PSA) testing – to model incidence trends in the absence of PSA testing. We used the assumptions of Mistry *et al* (2011) that PSA testing reached a steady state in 2004, and would continue at this level. To estimate the impact PSA testing had in 2004–2014, we first predicted these rates in the absence of any PSA testing (from projections based on 1979–1991 data). We then used these predicted rates to calculate age-specific observed/predicted ratios. We divided case numbers from 2004 to 2014 by these ratios to estimate cases in the absence of PSA testing. The projections for 2015–2035 were made by fitting the APC model to the 1975–1991 data, and also the modified data set from 2004 to 2014 and multiplying the model projections for 2004–2035 by the previously calculated observed/predicted ratios.

We used an age-stratified approach for breast cancer whereby we used data from before screening was offered to that particular age group of women (50–64 years during 1989–1996, 65–69 years during 1990–1997 and 2003–2014, and 70–74 years during 2004–2014) to estimate the rates when the screening programme reached a steady state in the specific age group. The observed/predicted ratio was used to adjust subsequent data from when the screening programmes were in place to make the projections until 2035. The projections for 2015–2035 were then multiplied by these observed/predicted ratios.

The ‘all cancers' numbers for incidence cases, and mortality deaths were compiled by summing the 26 cancers types and ‘other' cancer categories for each sex, following modelling these individually. The model predicts the number of cases or deaths. We converted this into incidence or mortality rates by dividing the projected number of cases or deaths by the population for each age band, and multiplying this by 100 000. Age-standardised rates (ASRs) for incidence and mortality were generated by performing weighted means using the European Standard Population (ESP) 2013. ASRs were calculated by age group, sex and site.

## Results

### Incidence

#### Projected rates

**Figure 1:**
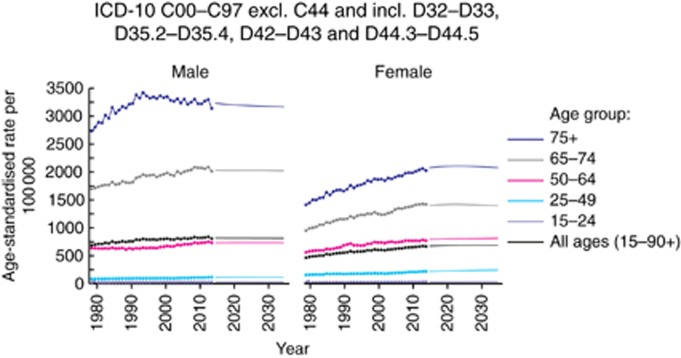
**Observed and projected incidence age-standardised rates (ASRs) per 100 000 15–90+ year olds, for all cancers combined by age group and sex.** Please note that projections for 1979–2014 are not shown due to modified data sets being used to calculate projections for breast and prostate cancers. For more details, please see ‘Materials and Methods' section.

Figure 1 displays observed trends (1979–2014, denoted by dots) and projections (2015–2035, denoted by a solid line) of age-standardised cancer incidence rates for males and females separately split down by age groups (all ages, 15–24, 25–49, 50–64, 65–74, 75+) for all cancer sites combined.

Figures for trends and projections by cancer site can be seen in Supplementary Material C. Cancers with similar incidence are grouped together so that *y* axes are comparable. We used log likelihood to assess model fit (see Supplementary Material D).

Projected data from 2015 to 2035 suggests that overall incidence ASR will increase by an average annual percentage of 0.07%, which corresponds to average annual decrease in males of 0.03%, and an increase in females of 0.11% (see Table 1).

Table 1 demonstrates that these changes in ASR for all cancers belies a complex pattern of increases and decreases in specific cancer types. In terms of average annual percentage change, thyroid cancer is the fastest accelerating cancer (males: 2.49%, females: 2.34%). Other cancers that are projected to accelerate quickly include oral cancer (males: 1.10%, females: 1.15%), kidney cancer (males: 1.08%, females: 0.70%), liver cancer (males: 1.41%, females: 0.52%) and anal cancer (males: 0.62%, females: 1.92%). For females only, large average annual percentage changes in cervical cancer (1.65%) incidence are projected. More modest increases are projected for Hodgkin lymphoma (males: 0.52%, females: 0.14%) and malignant melanoma (males: 0.26%, females: 0.29%).

Table 1 also demonstrates that for some cancers, opposite patterns of increasing and decreasing average annual percentage change in incidence rates are found between males and females. These include bone (males: −1.91%, females: 0.85%), leukaemia (males: 0.13%, females: −0.16%), oesophagal cancer (males: −0.31%, females: 0.02%), as well as the ‘other' cancer category (males: 0.22, females: −0.13%). For the remaining cancers, average annual percentage changes in incidence ASR were either small or constant.

#### Age-specific trends in incidence rates

Inspection of Supplementary Material C demonstrates that for the majority of cancers, incidence is higher for those in the 75+ age group. As such, changes in incidence ASRs are often a result of large changes in this age group, whereas the other age groups remain relatively constant. However, some cancers do not follow this pattern. For male and female thyroid cancer, increases were seen in all age groups. For males, the 65–74 age group is projected to increase higher than the 75+ age group; and for females, the 65–74, 50–64 and 25–49 age groups are all projected to rise higher than the 75+ age group. Similarly, for male oral cancer, the 65–74 age group is projected to increase more than the oldest age group. For some cancers, there is evidence of differing trends between the age groups. Overall increases are projected for ovary cancer due to increases in the 50–64 and 65–74 age groups, however, rates are projected to decrease in the 75+ age group. Similarly, substantial reductions in cervix cancer are projected for the 75+ age group, however, the overall increase is driven by changes in the 25–49 and 50–64 age groups. Notably for Hodgkin lymphoma, the youngest age group is projected to have the highest incidence over the period.

#### Projected cases

The total increase in cancer cases between 2014 and 2035 is 42.47% (corresponding to an average annual increase of 1.63%). Table 1 demonstrates that this increase in cancer cases is disproportionately projected for males (48.42%) compared with females (36.41%). These massive changes in the absolute number of cancer cases forecasted for the period 2014–2035 are mainly a result of the growing and ageing population.

Table 1 demonstrates that there are a number of cancers where large increases in the average annual percentage change in the number of cases for both males and females were projected. These include anal cancer (males: 2.01%, females: 3.09%), kidney cancer (males: 2.75%, females: 2.17%), leukaemia (males: 2.11%, females 1.52%), liver cancer (males: 3.28%, females: 2.38%), myeloma (males: 2.35%, females: 1.90%), oral cancer (males: 2.22%, females: 2.37%), pancreas cancer (males: 2.19%, females: 1.91%), stomach cancer (males: 0.89%, females: 1.30%) and thyroid cancer (males: 3.42%, females: 2.88%). For males only, prostate cancer had a substantial average annual percentage increase in cases at 2.34%. Mesothelioma cases are projected to decrease over the period 2015–2035 (males: −1.42%, females: −1.17%) per year on average. Bone cancer cases are also projected to decrease for males (−0.84%) over this period, but to increase for females (1.77%). All other cancers had relatively smaller average annual percentage increases.

#### Cancer incidence: past, present and future

**Figure 2:**
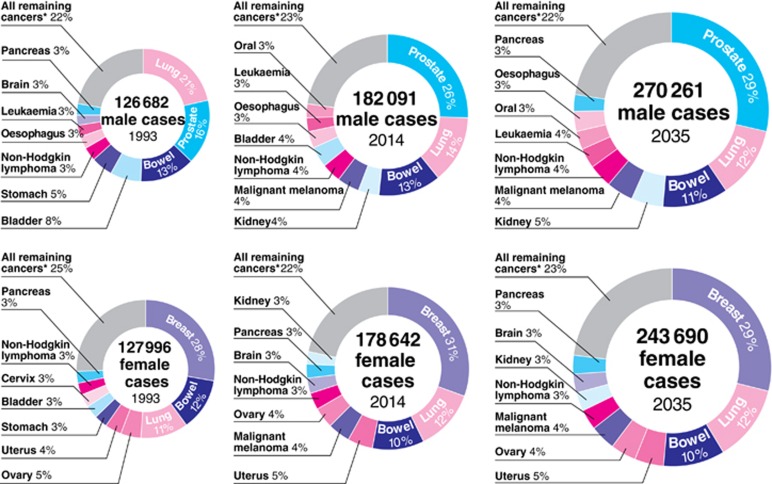
**Proportion of total cancer cases by cancer site in 1993 (observed), 2014 (observed) and 2035 (projected), split by sex.** The size of each doughnut is scaled to reflect the total number of cases. *All cancers (C00–C97 excluding C44) not otherwise individually named, plus D32–D33, D35.2–D35.4, D42–D43 and D44.3–D44.5 where brain is not individually named.

Figure 2 demonstrates the proportions of different cancer cases that make up the cancer population in 1993, 2014 and 2035. The size of the doughnut is scaled to reflect the total number of cancer cases in that year. For females, proportions of different cancers remain stable over time, with breast having the greatest proportion of cases for each of these years. In the most recent data available in 2014, lung cancer replaces bowel cancer as the second most common cancer, a trend which is set to continue until 2035. The trend for uterus cancer being a more common cancer is projected to continue.

In contrast for males, Figure 2 demonstrates that there are noticeable decreases in the proportion of lung cancer cases over the period. Replacing lung cancer in 1993, prostate cancer has become the most common cancer in men in 2014, and this is projected to continue until 2035. For bladder cancer, there is a trend for a decreasing proportion of cases with time. Conversely, kidney cancer and malignant melanoma are showing an increase in the proportion of cases, and this is projected to continue.

### Mortality

#### Projected rates

**Figure 3:**
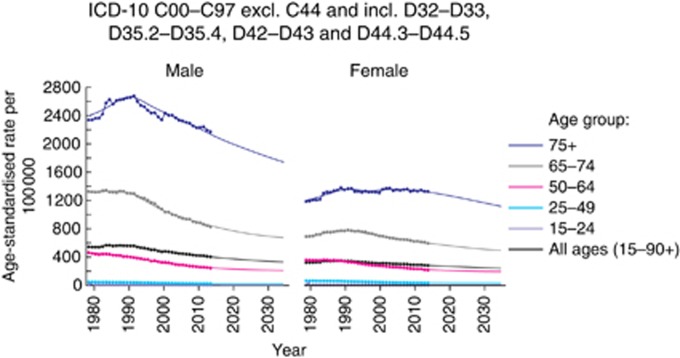
**Observed and projected mortality age-standardised rates (ASRs) per 100 000 15–90+ year olds, for all cancers combined by age group and sex.**

Figure 3 displays trends (1979–2014, denoted by dots) and projections (1979–2035, denoted by a solid line) of age-standardised cancer mortality rates for males and females separately split down by age groups (all ages, 15–24, 25–49, 20–64, 65–74, 75+) for all cancers combined. Overall, the mortality ASR will fall 15.30% between 2014 and 2035, with males declining by 18.33% and females by 14.57%.

Supplementary Material E shows this information for each cancer site. We used log likelihood to assess model fit (see Supplementary Material D).

We calculated the average annual percentage change in mortality ASR (see Table 2). The cancers with the fastest accelerating average annual increases in mortality rates are liver cancer (males: 1.99%, females: 1.79%), oral cancer (males: 1.42%, females: 1.53%) and anal cancer (males: 1.81%, females: 2.28%), and bone cancer for females (0.79%). More modest increases in average annual percentage change in mortality ASR are also noted for thyroid cancer in females (0.52%), though this is decreasing slightly in males (−0.27%). For females, there are increases in average annual percentage change mortality ASR in uterine cancer (0.73%) and laryngeal cancer (0.77%).

For all other cancers, the mortality rates are either relatively constant, or projected to decrease between 2015 and 2035 (see Table 2). The largest projected decrease in average annual percentage change in mortality ASR is in mesothelioma (males: −3.54%, females: −2.41%).

#### Age-specific trends

For the majority of cancers, the overall trend is driven by changes in the 75+ age group, which is largely due to the incidence burden being the highest among this age group.

It was noted that incidence rates of cervical cancer were rising most sharply in the 25–49 and 50–64 age groups, whereas a decline in incidence rates are projected for the 75+ age group. However, the mortality data displayed in Supplementary Material E suggest that the mortality rate is decreasing in all age groups, despite the increases in incidence noted.

Supplementary Material E also shows that for liver cancer in males, as well as the 75+ age group, the 50–64 and 65–74 age groups are contributing to the overall projected increases in mortality ASR. A similar pattern is noted for oral cancer and anal cancer, where these younger groups also contribute to the overall increases in mortality ASR.

#### Projected deaths

The number of cancer deaths is projected to increase 30.06% between 2014 and 2035. Table 2 demonstrates that this overall percentage obscures the vast differences between the genders, with a total increase in males of 35.04% and 24.48% in females. These increases are largely driven by shifting population demographics, as the opposite trends tend to be observed in mortality ASRs.

Large average annual percentage increases in deaths are predicted from liver cancer (males: 4.03%, females 3.76%), in anal cancer (males: 3.67%, females 3.75%), in oral cancer (males: 2.97%, females 3.09%), in pancreatic cancer (males: 2.06%, females: 1.58%) and in thyroid cancer (males: 1.91%, females 2.35%). For males, prostate cancer deaths are projected to increase by an average of 2.38% per year; and for females, uterine cancer deaths are projected to increase by 2.61% per year, on average. Mesothelioma is the only cancer where the annual average number of deaths is projected to decrease for both sexes (males −0.90%, females −0.21%). For males only, a reduction in the average annual number of deaths is projected for bone (−0.07%) and testicular (−0.79%) cancer. For females only, a reduction in Hodgkin lymphoma (−0.61%) and ovarian cancer (−0.48%) were projected over this period.

#### Cancer mortality: past, present and future

**Figure 4:**
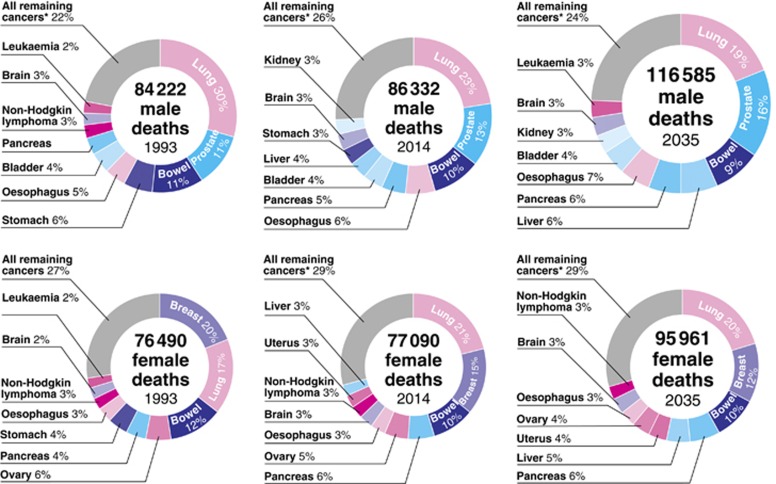
**Proportion of total cancer deaths by cancer site in 1993 (observed), 2014 (observed) and 2035 (projected), split by sex.** The size of each doughnut is scaled to reflect the total number of deaths.

Figure 4 demonstrates the proportions of cancer deaths in 1993, 2014 and 2035. The size of the doughnut is scaled to reflect the total number of cancer deaths in that year. For women, as a consequence of lung cancer having increased incidence, it is the most common cancer death among women in 2014, and this trend is projected to continue to 2035. From 2014 to 2035, uterine cancer is projected to increase from the ninth most common cancer death in women, to the sixth, which again reflects the increased incidence of uterine cancer.

For men, lung cancer is the most common cancer death throughout this period. Stomach and bladder cancer become less common causes of cancer death between 1993 and 2014, and this is projected to decrease even further. For both pancreatic and liver cancer, the proportion of deaths attributable to these cancer types is projected to rise over this period. Incidence rates for both these cancers is increasing, and here they are projected to increase even more between 2015 and 2035, which means, in the context of relatively little treatment improvements for these cancers, we can expect a greater proportion of cancer deaths to be as a result of these cancers.

## Discussion

Here we show projections of cancer incidence rates until 2035 demonstrating a small increase for females, and a very slight decrease for males. We have projected that overall mortality rates for both males and females will decline over the same period. The overall number of cancer cases and deaths will increase substantially over this period, which is largely a result of the increasing population size and the ageing population.

Notably, our findings contrast to the findings of Mistry *et al* (2011), who report a gradual levelling off of the incidence ASR for all cancers combined, with rates falling by 1% in males and 1.9% in females. Mistry *et al* (2011) used the European standardised population from 1976 (Waterhouse *et al*, 1976), whereas we have used an updated version for 2013 (Eurostat, 2013). As the ESP 2013 gives older age categories more weight than the ESP 1976, this will amplify the incidence and mortality rates as cancer is disproportionately diagnosed in older age groups. We have compared the impact of using the ESP 2013 and ESP 1976 (Supplementary Materials F, F.1 and F.2). This demonstrates that although using the ESP 1976 for age-standardisation results in lower rates for the majority of cancers, trends seen when using ESP 2013 are similar to those observed when using ESP 1976. This suggests that there are genuine increases in rates, and this is not simply an artefact of the different age-standardisation weights. There have been recent increases in the prevalence of risk factors associated with cancer such as being overweight and obese (Health and Social Care Information Centre, 2012), which may explain these increases in incidence rates.

Incidence data sets have year-on-year changes owing to late registrations. These have previously been shown to cause an artificial downward trend in incidence for the most recent years (Oliver *et al*, 2013). In Supplementary Materials G, G.1 and G.2, we examine the extent of late registration in cancer registration data sets between 2000 and 2014. Both bone cancer and leukaemia show a discrepant result between males and females, where incidence ASR is decreasing in males and increasing in females. As there are no risk factors strongly associated with the development of either of these cancers, it is difficult to explain these patterns. However, as highlighted in Supplementary Material G.2, these cancers are the most affected by the late-registration problem, which may artificially decrease the rates. As a result of this potential data quality issue, firm conclusions cannot be drawn for these cancers from the pattern of projections presented here.

We did not create modified data sets for either bowel or cervical cancer, although there are screening programmes for these cancers. Besides early detection of cancer, these screening programmes are a means of primary prevention through the identification and removal of pre-cancerous lesions. The cervical screening programme has been established since 1988 and its benefits on incidence and mortality are evident in the data, and therefore will be reflected in the projections. Previous modelling research on the bowel screening programme suggests the programme will cause incidence rates to increase until 2017, following which incidence will begin to decrease (Parkin *et al*, 2008). As such, there is no benefit in creating modified data sets to model the underlying trends in incidence rates in the absence of the additional cases resulting from screening activity, as it is intended that the screening will affect these underlying rates. Bowel cancer projections should therefore be interpreted with caution. Future bowel cancer incidence may be overestimated in the projections, as typically there is a prevalence wave following the introduction of a screening programme. Owing to the time lag between screening benefits and its impact on mortality, it is likely the current mortality data set reflects little, if any, benefit of the screening programme, and therefore these mortality projections may provide a reference point to evaluate the effectiveness of the screening programme.

Increases in incidence and mortality have been noted for cancers associated with the human papilloma virus (HPV), including oral, anal and cervical cancer. However, the benefits of the HPV vaccination programme have not yet been realised. The vaccination programme was introduced throughout UK in 2008, offering the vaccine to 12–13-year-old girls. Therefore these projections could act as a benchmark to evaluate the efficacy of the vaccine, as its impact is not yet reflected in the data.

Liver cancer incidence and mortality is projected to increase. There are numerous risk factors associated with the development of liver cancer including obesity, alcohol and the hepatitis infection (Parkin *et al*, 2011). However, there is poor concordance between initial diagnosis of liver cancer and death certificate information for liver cancer (Lund *et al*, 2010). This may be because the liver is a frequent site of metastasis, so many cancer deaths are recorded as liver cancer deaths even when this is not the primary site. This may mean that this is an artefactual increase in mortality. However, given that liver cancer incidence is increasing and survival for this cancer is relatively poor, it is possible this is a genuine increase in liver cancer mortality.

Dramatic increases are projected for thyroid cancer, however, there are no established risk factors for this cancer. Overdiagnosis of thyroid cancer is often cited as a reason for the increases in incidence rates observed for this disease (Lee and Shin, 2014). However, here we additionally noted that for females, there are small projected increases in mortality ASR. This may suggest that overdiagnosis alone cannot explain all of the observed increases in thyroid cancer.

It is also evident in the data and projections that men disproportionately bear the burden of cancer. Despite the recent observed increases in females, females are not projected to outstrip men either in terms of incidence or mortality during this period. This gender imbalance should be considered by public health professionals when targeting interventions aimed at reducing the burden of cancer.

There are assumptions and limitations associated with the approach we have taken here. Supplementary Material B details the process by which we selected the link function, geometric dampening and the number of knots for each of the age, period and cohort components. We assumed that the model that was able to most accurately project the data over the period 1999–2014 would provide the most accurate projections. The basis of the APC model is that past trends will be continued into the future; however, the pace of change over the coming years may be such that another model would have been more appropriate. Furthermore, the development of vaccines for certain cancers, or the development of radical curative treatments could result in a drastic change in cancer incidence and mortality, respectively. Our model does not anticipate these profound changes. Therefore it is important that projections are completed at regular intervals in order that the most recent trends in the data can be captured. Different modelling approaches, which attempt to explicitly model changes for cancers where large breakthroughs in either treatment or prevention are anticipated, would be a useful complement to this current work.

Despite attempts to optimise this model, there will still be error associated with these projections, and we have presented a projection interval in Supplementary Materials H, H.1 and H.2, which attempts to quantify the extent of this uncertainty. Although these projections are useful to provide estimate to healthcare planners regarding what the future burden of the cancer population will be, it is likely that these projections will deviate from actual numbers the further into the future you look. Projections completed at regular intervals, which incorporate the most recent trends will help to minimise the error associated with future projections of cancer burden.

Here we show projections of cancer incidence and mortality until 2035. Projections of incidence demonstrate that the massive efforts to reduce smoking prevalence should be continued, as lung cancer still constitutes a large proportion of the cancer population. In addition, greater efforts are required to tackle other risk factors such as alcohol, overweight and obesity, the hepatitis infection and HPV, as the incidence of numerous cancers linked to these risk factors is also set to increase. Finally, the projected number of cases and deaths demonstrates the massive burden that cancer will be, which should be planned for accordingly.

## Figures and Tables

**Table 1:** Incidence age standardised rates (ASRs) per 100 000 15–90+ year olds, cases, total percentage changes and average annual percentage changes (AAPC) for 1993 (observed), 2014 (observed) and 2035 (projected) by cancer site and sex

	**1993 ASR**	**2014 ASR**	**2035 ASR**	**ASR % change 2014–2035**	**ASR AAPC 2015–2035**	**1993 Cases**	**2014 Cases**	**2035 Cases**	**Cases % change 2014–2035**	**Cases AAPC 2015–2035**
**Males**										
**Cancer site**										
Anus	1.73	1.84	2.18	18.24	0.62	284	434	684.68	57.76	2.01
Bladder	61.52	33.92	21.07	−37.88	−2.33	9705	7301	7531.11	3.15	0.16
Bone	0.91	1.01	0.69	−31.35	−1.91	189	256	216.77	−15.33	−0.84
Bowel	105.04	102.38	86.92	−15.10	−0.86	16 929	22 827	29 356.10	28.60	1.13
Brain	17.41	21.08	22.11	4.87	0.14	3364	5036	6884.30	36.70	1.40
Hodgkin lymphoma	3.50	4.48	4.87	8.86	0.52	810	1148	1479.68	28.89	1.27
Kidney	19.12	34.18	43.88	28.38	1.08	3275	7778	14 258.84	83.32	2.75
Larynx	10.91	8.36	6.52	−22.04	−1.19	1902	1920	2099.54	9.35	0.41
Leukaemia	21.22	24.14	25.59	6.00	0.13	3461	5425	8714.08	60.63	2.11
Liver	6.36	16.21	23.23	43.27	1.41	1043	3636	7769.55	113.68	3.28
Lung	167.74	112.25	97.06	−13.54	−0.70	27 112	24 767	32 874.81	32.74	1.34
Malignant melanoma	11.99	33.28	35.42	6.46	0.26	2248	7715	11 897.43	54.21	2.03
Mesothelioma	6.05	10.54	4.75	−54.98	−4.07	1051	2289	1753.23	−23.41	−1.42
Myeloma	10.93	13.76	15.52	12.76	0.39	1729	3072	5228.66	70.20	2.35
Non-Hodgkin Lymphoma	23.02	32.45	31.56	−2.75	−0.23	4068	7440	10 489.40	40.99	1.54
Oesophagus	23.95	26.67	25.07	−6.00	−0.31	3911	6019	8365.78	38.99	1.57
Oral	13.27	21.38	28.40	32.84	1.10	2323	5124	8675.81	69.32	2.22
Pancreas	20.74	21.71	23.18	6.75	0.18	3321	4833	7874.14	62.92	2.19
Prostate	136.51	208.01	232.54	11.79	0.48	20 065	46 689	77 348.83	65.67	2.34
Stomach	42.75	19.80	15.27	−22.90	−1.24	6739	4353	5220.27	19.92	0.89
Testis	6.56	8.88	9.97	12.30	0.55	1639	2406	2920.80	21.40	0.91
Thyroid	1.59	3.91	6.93	77.43	2.49	310	964	2089.44	116.75	3.42
Other	70.47	47.87	49.78	3.98	0.22	11 204	10 659	16 527.69	55.06	2.11
All cancer sites	783.29	808.13	812.52	0.54	−0.03	126 682	182 091	270 260.90	48.42	1.82
**Females**										
**Cancer site**										
Anus	1.61	3.27	5.11	56.34	1.92	360	873	1748.81	100.32	3.09
Bladder	16.93	9.94	6.87	−30.95	−1.87	3881	2756	2855.27	3.60	0.11
Bone	0.69	0.86	1.06	24.16	0.85	169	235	351.39	49.53	1.77
Bowel	68.77	67.67	63.22	−6.58	−0.34	15 848	18 401	24 289.91	32.00	1.32
Brain	14.10	20.44	21.97	7.50	0.30	3233	5489	7397.15	34.76	1.35
Breast	160.46	204.93	209.51	2.24	0.14	35 585	54 833	71 022.04	29.52	1.26
Cervix	16.28	11.81	16.85	42.68	1.65	3805	3223	4792.35	48.69	1.86
Hodgkin lymphoma	2.17	3.21	3.17	−1.05	0.14	553	881	993.46	12.77	0.77
Kidney	8.51	17.27	20.42	18.21	0.70	1927	4634	7473.54	61.28	2.17
Larynx	2.12	1.48	1.45	−2.14	−0.43	469	389	498.93	28.26	0.81
Leukaemia	11.83	13.15	13.01	−1.10	−0.16	2753	3566	5043.55	41.43	1.52
Liver	3.02	6.87	8.32	21.02	0.52	692	1884	3363.86	78.55	2.38
Lung	62.61	80.94	80.46	−0.59	−0.12	14 177	21 633	29 957.38	38.48	1.44
Malignant melanoma	14.54	28.64	30.40	6.14	0.29	3374	7698	10 277.73	33.51	1.37
Mesothelioma	0.80	1.60	0.86	−45.98	−3.42	178	428	362.64	−15.27	−1.17
Myeloma	7.19	9.00	9.61	6.76	0.25	1644	2428	3659.35	50.71	1.90
Non-Hodgkin lymphoma	16.01	22.67	21.92	−3.32	−0.36	3651	6069	8131.89	33.99	1.21
Oesophagus	11.75	10.62	10.77	1.44	0.02	2730	2900	4291.51	47.98	1.81
Oral	5.52	9.61	12.71	32.24	1.15	1243	2556	4384.60	71.54	2.37
Ovary	27.22	27.67	31.89	15.23	0.61	6064	7367	10 500.59	42.54	1.58
Pancreas	15.47	17.60	18.38	4.47	0.12	3573	4783	7282.64	52.26	1.91
Stomach	17.66	8.48	7.72	−8.92	−0.47	4136	2329	3061.72	31.46	1.30
Thyroid	3.42	9.01	15.68	74.07	2.34	815	2424	4749.60	95.94	2.88
Uterus	21.84	35.51	33.09	−6.84	−0.39	4810	9323	11 576.13	24.17	0.95
Other	53.26	42.00	40.89	−2.62	−0.13	12 326	11 540	15 623.74	35.39	1.45
All cancer sites	563.75	664.27	685.37	3.18	0.11	127 996	178 642	243 689.80	36.41	1.43

**Table 2:** Mortality age standardised rates (ASRs) per 100 000 15–90+ year olds, deaths, total percentage changes, and average annual percentage changes (AAPC) for 1993, 2014 and 2035 by cancer site and sex

	**1993 ASR**	**2014 ASR**	**2035 ASR**	**ASR Percentage Change 2014–2035**	**ASR AAPC 2015–2035**	**1993 deaths**	**2014 deaths**	**2035 deaths**	**Deaths Percentage Change 2014–2035**	**Deaths AAPC 2015–2035**
**Males**										
**Cancer site**										
Anus	0.62	0.66	0.91	37.90	1.81	94	143	302.52	111.55	3.67
Bladder	25.66	17.98	13.79	−23.31	−1.19	3593	3614	5189.82	43.60	1.78
Bone	0.63	0.80	0.48	−40.29	−1.49	128	187	154.71	−17.27	−0.07
Bowel	60.00	40.41	28.86	−28.58	−1.59	9155	8566	10270.28	19.90	0.87
Brain	11.55	12.22	11.97	−2.10	−0.19	2125	2821	3887.01	37.79	1.43
Hodgkin Lymphoma	1.18	0.91	0.75	−18.09	−0.39	235	211	250.45	18.70	1.35
Kidney	10.65	12.75	11.40	−10.59	−0.47	1752	2771	3957.66	42.82	1.74
Larynx	4.66	3.10	2.80	−9.77	−0.39	761	677	954.03	40.92	1.67
Leukaemia	12.81	12.41	9.64	−22.35	−1.32	2041	2630	3551.68	35.04	1.32
Liver	6.68	13.85	21.75	57.01	1.99	1106	3052	7449.34	144.08	4.03
Lung	155.34	89.69	64.97	−27.56	−1.45	24 910	19 563	22226.74	13.62	0.69
Malignant Melanoma	4.15	6.44	5.57	−13.38	−0.57	730	1431	1974.49	37.98	1.66
Mesothelioma	5.94	9.93	5.10	−48.66	−3.54	1030.20	2153.58	1875.83	−12.90	−0.90
Myeloma	7.85	7.50	6.33	−15.64	−0.69	1225	1596	2264.73	41.90	1.79
Non-Hodgkin Lymphoma	13.37	12.10	9.49	−21.58	−1.20	2245	2603	3434.57	31.95	1.27
Oesophagus	23.91	23.53	19.35	−17.79	−0.96	3876	5213	6570.96	26.05	1.09
Oral	6.19	6.97	9.51	36.54	1.42	1035	1613	3074.45	90.60	2.97
Pancreas	20.31	20.07	20.00	−0.36	−0.06	3208	4426	6871.66	55.26	2.06
Prostate	71.61	57.22	47.86	−16.36	−0.74	9519	11 287	18336.23	62.45	2.38
Stomach	33.48	13.69	9.31	−32.00	−1.80	5148	2919	3294.87	12.88	0.63
Testis	0.59	0.24	0.15	−34.87	−2.24	118	60	51.13	−14.79	−0.79
Thyroid	0.72	0.71	0.68	−4.63	−0.27	120	154	234.55	52.30	1.91
Other	64.78	40.75	29.22	−28.30	−1.68	10 068	8641	10407.62	20.44	0.81
All cancers	542.69	403.92	329.86	−18.33	−0.94	84222.20	86331.58	116585.30	35.04	1.45
**Females**										
**Cancer site**										
Anus	0.62	0.78	1.26	61.84	2.28	139	215	474.84	120.86	3.75
Bladder	7.47	6.12	5.78	−5.56	−0.29	1763	1755	2581.42	47.09	1.87
Bone	0.39	0.55	0.54	−2.67	0.79	96	152	197.29	29.80	2.15
Bowel	38.91	25.97	21.08	−18.83	−0.99	9019	7337	8976.63	22.35	1.04
Brain	8.08	8.47	8.26	−2.40	−0.21	1830	2295	3072.60	33.88	1.31
Breast	64.89	41.15	30.51	−25.86	−1.42	14 612	11 360	11875.54	4.54	0.26
Cervix	7.37	3.31	3.08	−6.90	−0.37	1681	890	1029.95	15.72	0.66
Hodgkin Lymphoma	0.70	0.52	0.31	−39.82	−2.21	170	143	117.70	−17.69	−0.61
Kidney	5.00	5.88	4.29	−27.07	−1.56	1145	1637	1781.37	8.82	0.44
Larynx	0.93	0.61	0.74	21.97	0.77	211	162	274.68	69.56	2.25
Leukaemia	7.66	6.82	5.78	−15.31	−1.02	1785	1907	2539.11	33.15	1.16
Liver	3.64	7.38	11.33	53.40	1.79	838	2035	4730.14	132.44	3.76
Lung	55.99	60.52	51.81	−14.38	−0.70	12 737	16 331	19604.32	20.04	0.92
Malignant Melanoma	3.51	3.71	3.05	−17.98	−0.88	802	1027	1235.89	20.34	1.01
Mesothelioma	0.67	1.59	1.01	−36.47	−2.41	149.62	423.87	422.05	−0.43	−0.21
Myeloma	5.45	4.79	3.72	−22.24	−1.18	1259	1332	1570.42	17.90	0.88
Non-Hodgkin Lymphoma	8.61	7.86	6.25	−20.49	−1.20	1998	2183	2694.18	23.42	0.96
Oesophagus	11.06	9.25	7.64	−17.44	−0.78	2587	2577	3176.99	23.28	1.18
Oral	2.37	2.82	4.00	41.82	1.53	535	773	1517.44	96.31	3.09
Ovary	19.23	15.36	9.62	−37.42	−2.28	4288	4127	3743.83	−9.28	−0.48
Pancreas	14.84	16.02	15.12	−5.63	−0.31	3428	4391	6132.70	39.67	1.58
Stomach	14.17	5.92	4.53	−23.62	−1.32	3342	1657	1897.55	14.52	0.67
Thyroid	1.06	0.79	0.93	17.21	0.52	248	222	377.76	70.16	2.35
Uterus	6.35	7.98	9.47	18.66	0.73	1453	2166	3829.07	76.78	2.61
Other	44.69	35.61	28.93	−18.76	−1.09	10 374	9992	12107.30	21.17	0.87
All cancers	333.68	279.78	239.02	−14.57	−0.77	76489.62	77089.87	95960.78	24.48	1.06

## References

[bib1] Bray F, Møller B (2006) Predicting the future burden of cancer. Nat Rev Cancer 6: 63–74. 16372017 10.1038/nrc1781

[bib2] Duffy SW, Nagtegaal ID, Wallis M, Cafferty FH, Houssami N, Warwick J, Allgood PC, Kearins O, Tappenden N, O'Sullivan E, Lawrence G (2008) Correcting for lead time and length bias in estimating the effect of screen detection on cancer survival. Am J Epidemiol 168: 98–104. 18504245 10.1093/aje/kwn120

[bib3] Ellis L, Woods LM, Estéve J, Eloranta S, Coleman MP, Rachet B (2014) Cancer incidence, survival and mortality: explaining the concepts. Int J Cancer 135(8): 1774–1782. 24945976 10.1002/ijc.28990

[bib4] Eurostat (2013) Available at: http://ec.europa.eu/eurostat/en/web/products-manuals-and-guidelines/-/KS-RA-13-028 accessed September 2015.

[bib5] Health and Social Care Information Centre Health Survey for England (2012) Available at: http://www.hscic.gov.uk/catalogue/PUB13218 (accessed April 2016).

[bib6] Lee JH, Shin SW (2014) Overdiagnosis and screening for thyroid cancer in Korea. Lancet 384: 1848. 25457916 10.1016/S0140-6736(14)62242-X

[bib7] Lund JL, Harlan LC, Yabroff KR, Warren JL (2010) Should cause of death from the death certificate be used to examine cancer-specific survival? A study of patients with distant stage disease. Cancer Invest 28: 758–764. 20504221 10.3109/07357901003630959 PMC3097383

[bib8] Mistry M, Parkin DM, Ahmad AS, Sasieni P (2011) Cancer incidence in the United Kingdom: projections to the year 2030. Br J Cancer 105: 1795–1803. 22033277 10.1038/bjc.2011.430 PMC3242594

[bib9] Møller B, Fekjaer H, Hakulinen T, Tryggvadóttir L, Storm HH, Talbäck M, Haldorsen T (2002) Prediction of cancer incidence in the Nordic countries up to 2020. Eur J Cancer Prev 11: 1–96. 12442806

[bib10] Møller B, Fekjaer H, Hakulinen T, Sigvaldason H, Storm HH, Talbäck M, Haldorsen T (2003) Prediction of cancer incidence in the Nordic countries: empirical comparison of different approaches. Stat Med 22: 2751–2766. 12939784 10.1002/sim.1481

[bib11] Oliver SE, Roman E, Crouch S, Bolton E, Ferguson B (2013) Comment on ‘cancer incidence in the United Kingdom: projections to the year 2030'. Br J Cancer 108: 1213–1214. 23429209 10.1038/bjc.2013.71 PMC3619081

[bib12] Olsen AH, Parkin DM, Sasieni P (2008) Cancer mortality in the United Kingdom: projections to the year 2025. Br J Cancer 99: 1549–1554. 18854832 10.1038/sj.bjc.6604710 PMC2579704

[bib13] ONS The impact of calculating mortality rates using the 2013 European Standard Population on causes of death. Available at: http://www.ons.gov.uk/ons/guide-method/user-guidance/health-and-life-events/revised-european-standard-population-2013—2013-esp-/rpt-2013-esp-mortality-rates.pdf (accessed December 2015).

[bib14] Parkin DM, Tappenden P, Olsen AH, Patnick J, Sasieni P (2008) Predicting the impact of the screening programme for colorectal cancer in the UK. J Med Screen 15: 163–174. 19106256 10.1258/jms.2008.008024

[bib15] Parkin DM, Boyd L, Walker LC (2011) 16. The fraction of cancer attributable to lifestyle and environmental factors in the UK in 2010. Br J Cancer 105: S77–S81. 22158327 10.1038/bjc.2011.489 PMC3252065

[bib16] Sasieni PD (2012) Age-period-cohort models in Stata. Stata J 12(1): 45–60.

[bib17] Sedjo RL, Byers T, Levin TR, Haffner SM, Saad MF, Tooze JA, D'Agostino RB (2007) Change in body size and the risk of colorectal adenomas. Cancer Epidemiol Biomarkers Prev 16: 526. 17372248 10.1158/1055-9965.EPI-06-0229

[bib18] Waterhouse J, Muir CS, Correa P, Powell J (eds) (1976) Cancer Incidence in Five Continents Vol. III: 456 pp IARC Scientific Publications No. 15.

[bib19] Welch HG, Black WC (2010) Overdiagnosis in Cancer. J Natl Cancer Inst 102: 605–613. 20413742 10.1093/jnci/djq099

