# Supplementary material for: Cancer incidence and mortality projections in the UK until 2035
Source: Br J Cancer. 2016 Oct 11;115(9):1147–55. doi: 10.1038/bjc.2016.304 (PMC5117795; doi:10.1038/bjc.2016.304)

Observed and projected incidence age standardised rates (ASRs) per 100,000 15-90+ year olds, for each cancer site by age group and sex

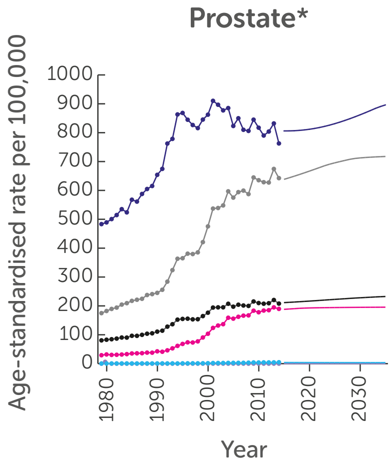

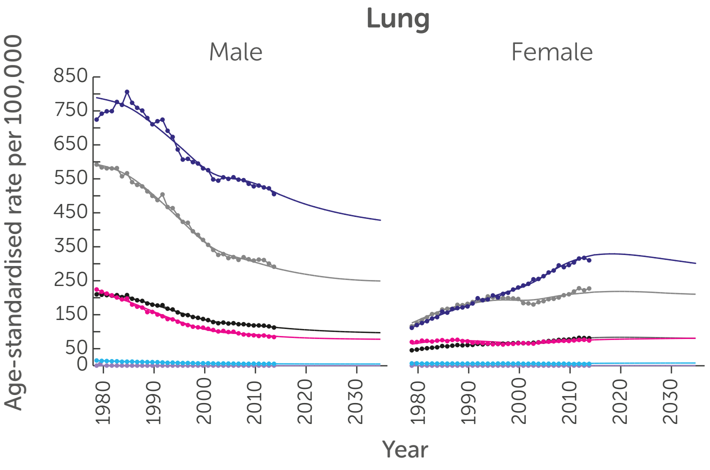

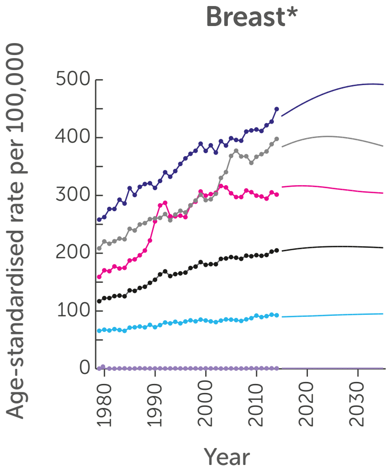

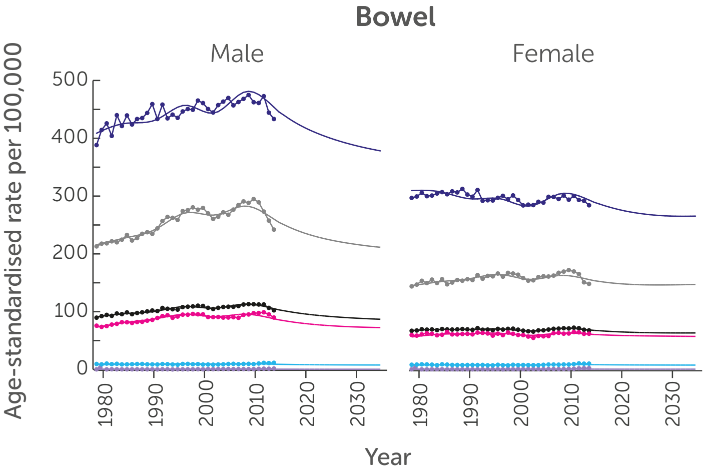

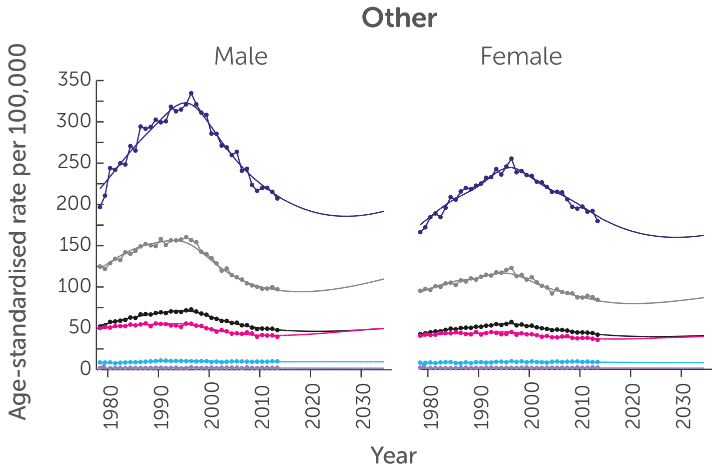

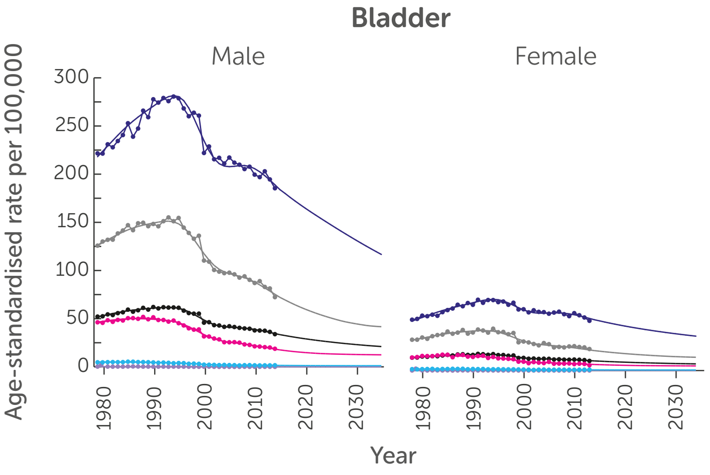

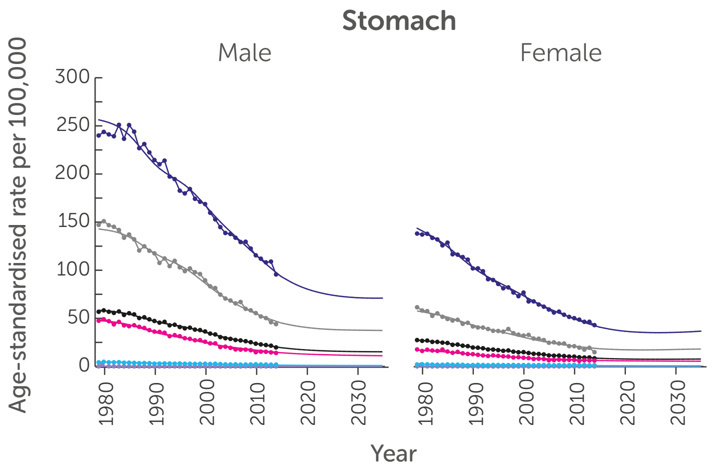

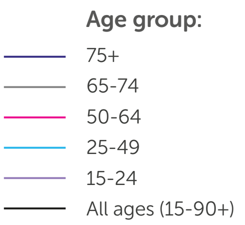

*Please note that projections for 1979-2014 are not shown due to a modified dataset being used to calculate projections for this cancer site. For more details, please see Materials and Methods.

Observed and projected incidence age standardised rates (ASRs) per 100,000 15-90+ year olds, for each cancer site by age group and sex

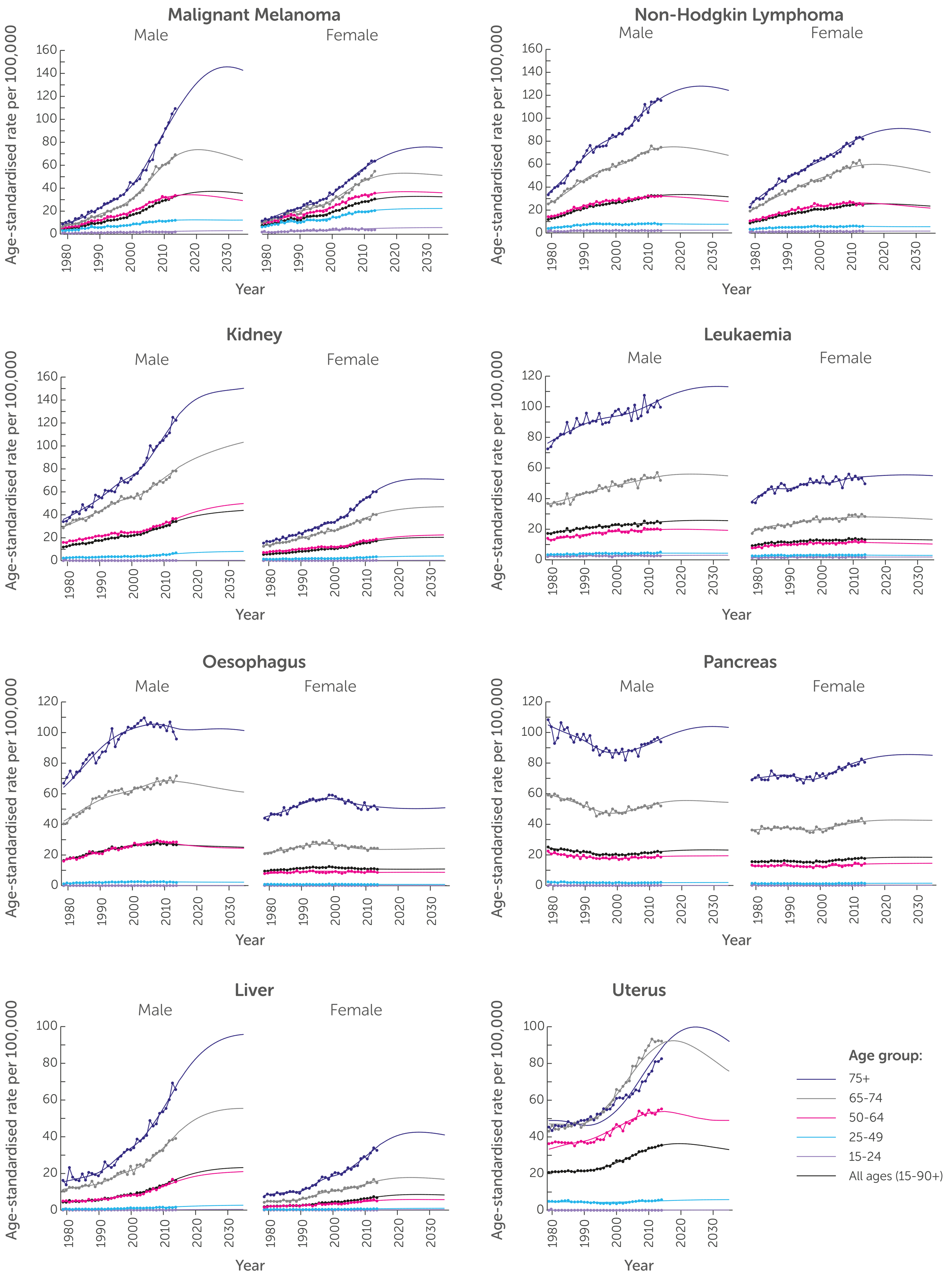

Observed and projected incidence age standardised rates (ASRs) per 100,000 15-90+ year olds, for each cancer site by age group and sex

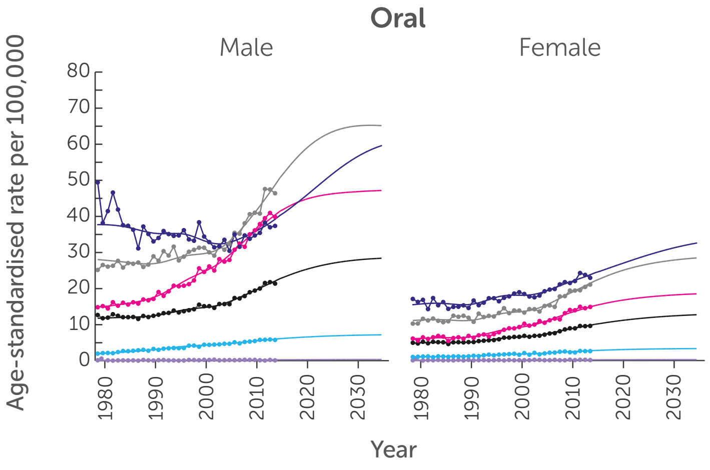

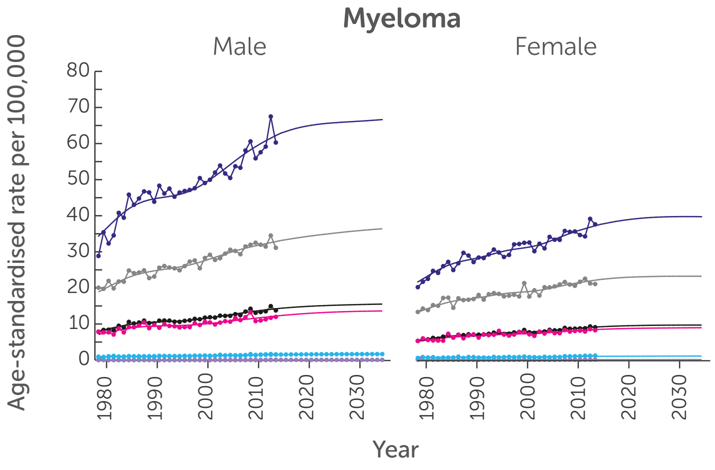

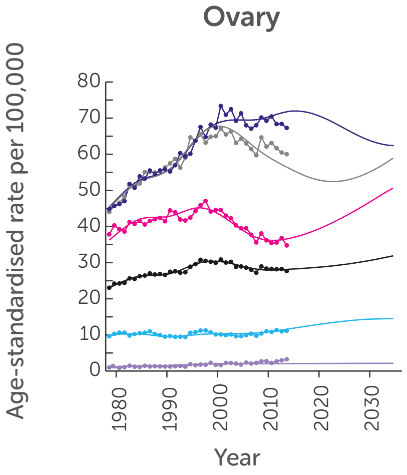

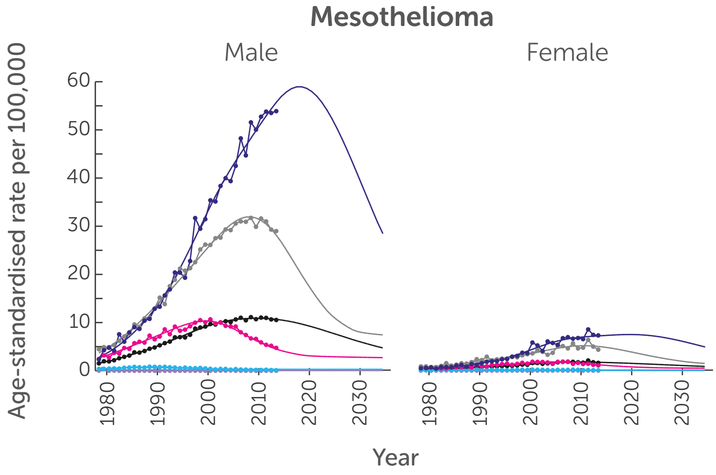

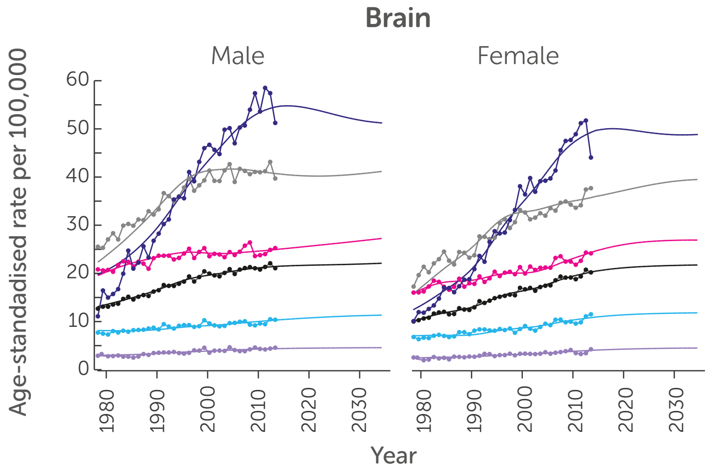

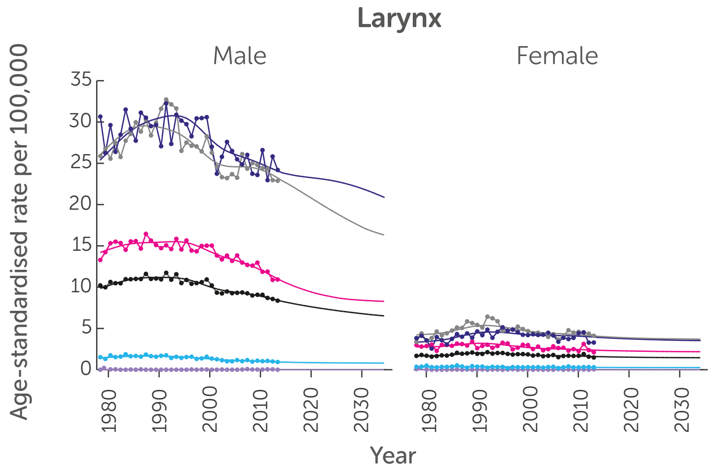

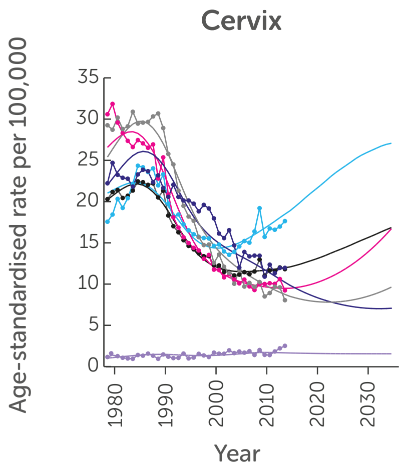

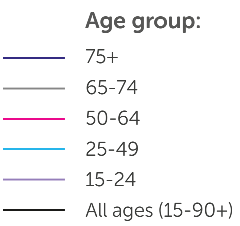

Observed and projected incidence age standardised rates (ASRs) per 100,000 15-90+ year olds, for each cancer site by age group and sex

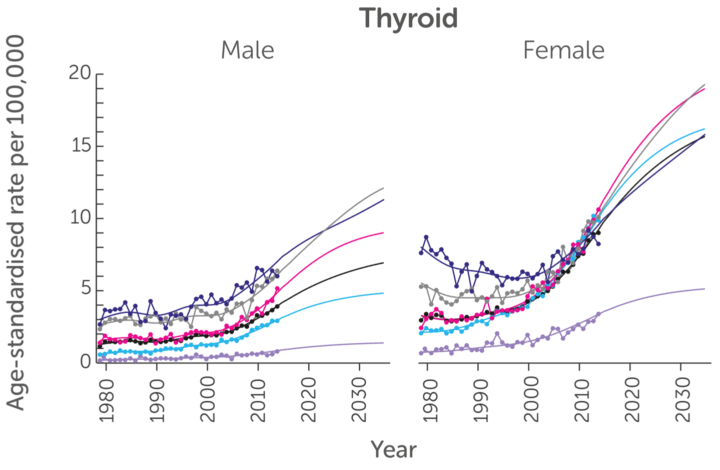

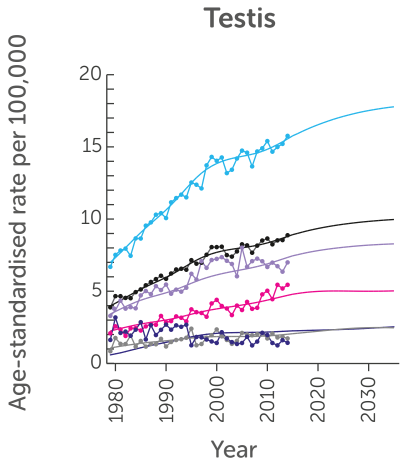

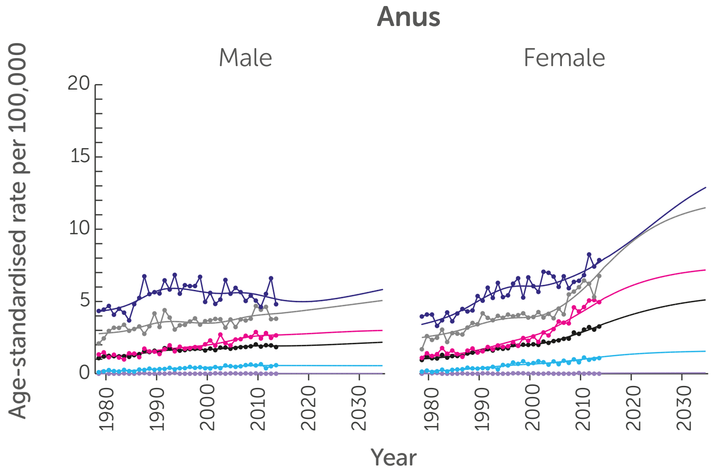

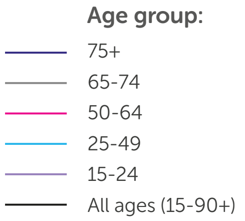

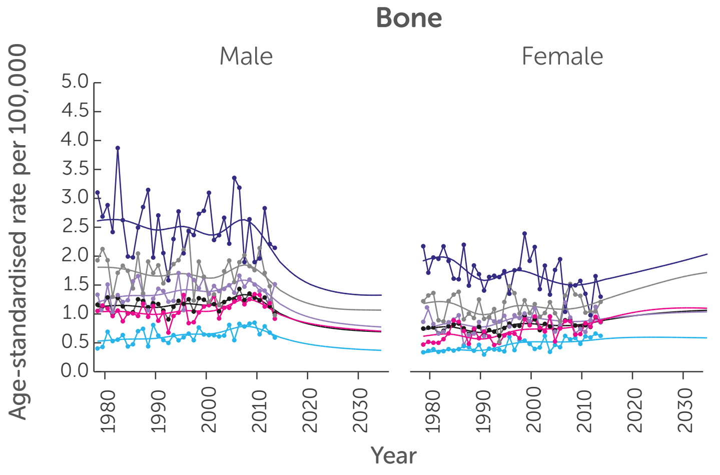

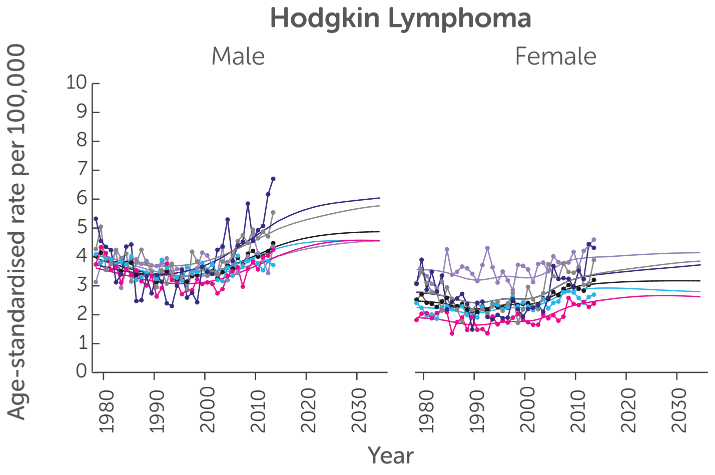

Supplement: Supplementary Material C [file bjc2016304x4.pdf]
