# Supplementary material for: Cancer incidence and mortality projections in the UK until 2035
Source: Br J Cancer. 2016 Oct 11;115(9):1147–55. doi: 10.1038/bjc.2016.304 (PMC5117795; doi:10.1038/bjc.2016.304)

Observed and projected mortality age standardised rates (ASRs) per 100,000 15-90+ year olds, for each cancer site by age group and sex

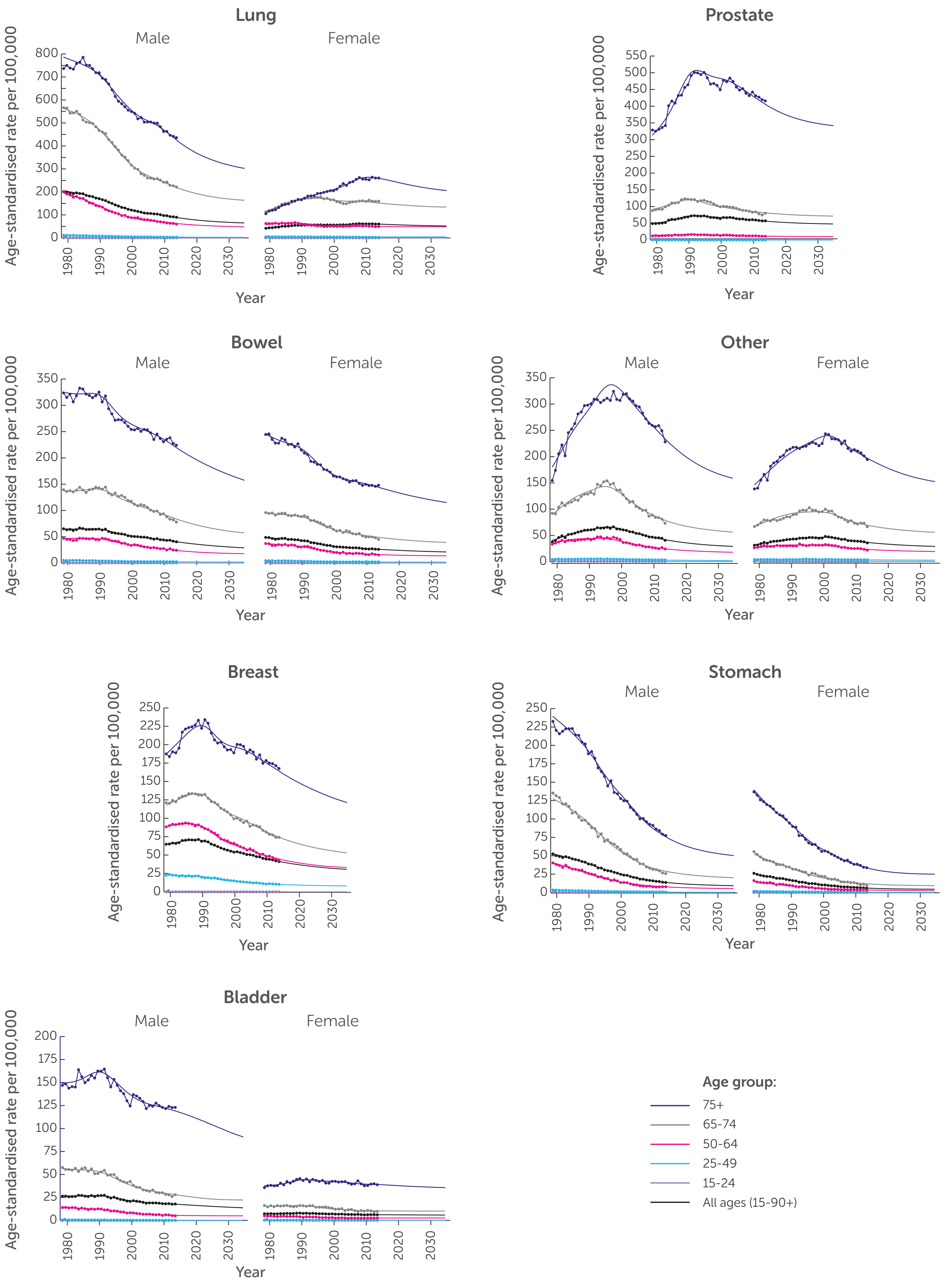

Observed and projected mortality age standardised rates (ASRs) per 100,000 15-90+ year olds, for each cancer site by age group and sex

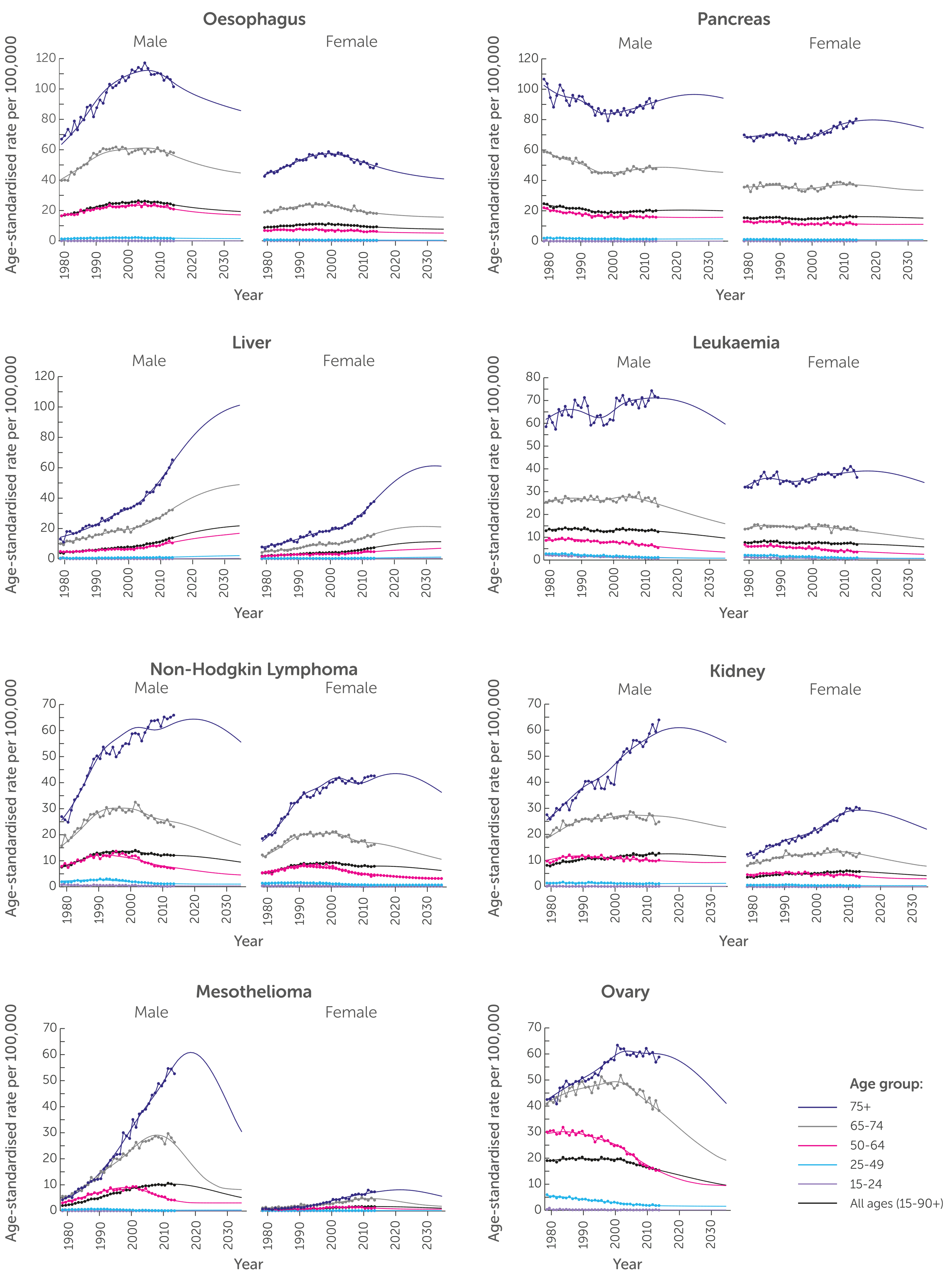

Observed and projected mortality age standardised rates (ASRs) per 100,000 15-90+ year olds, for each cancer site by age group and sex

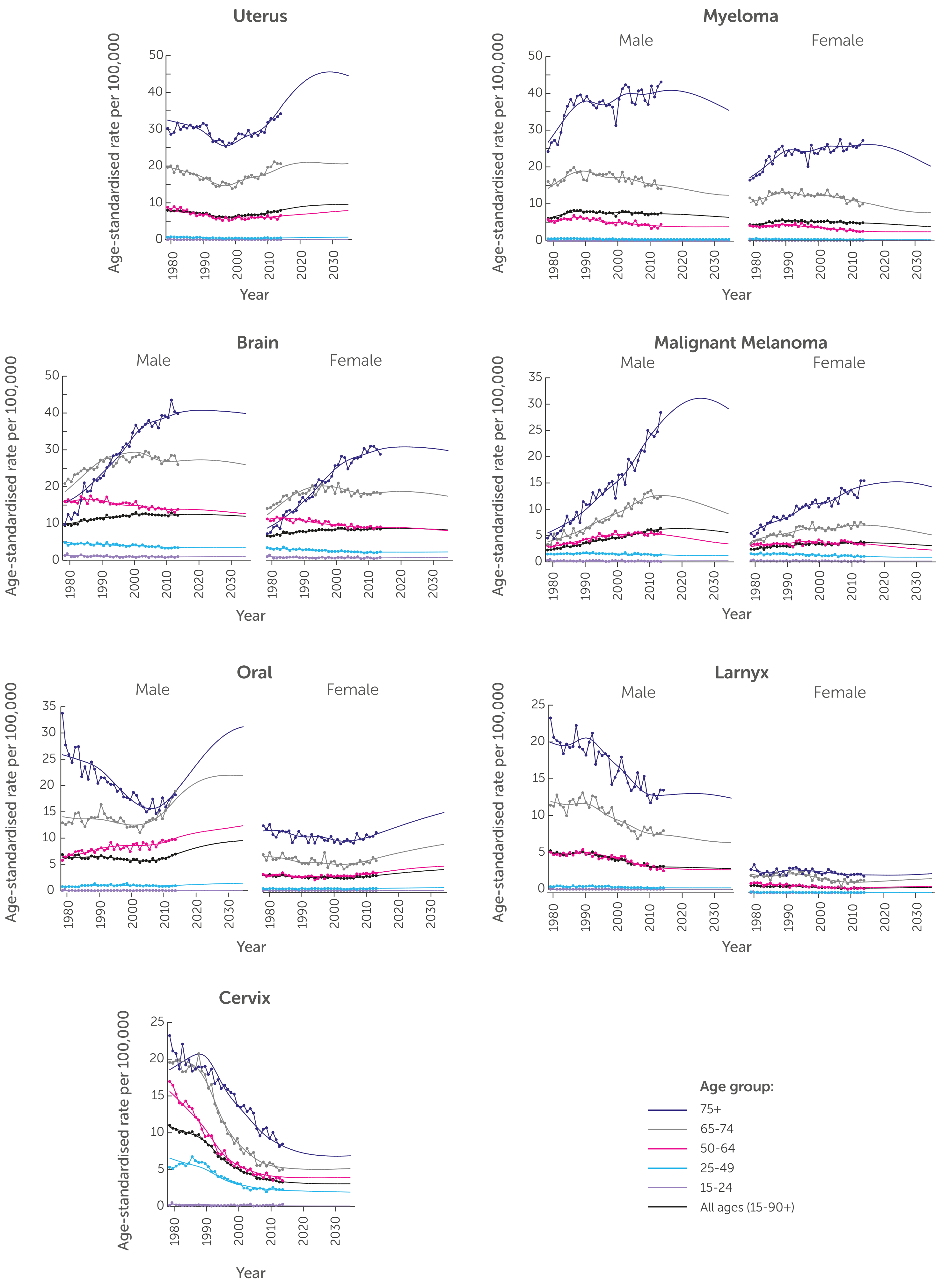

Observed and projected mortality age standardised rates (ASRs) per 100,000 15-90+ year olds, for each cancer site by age group and sex

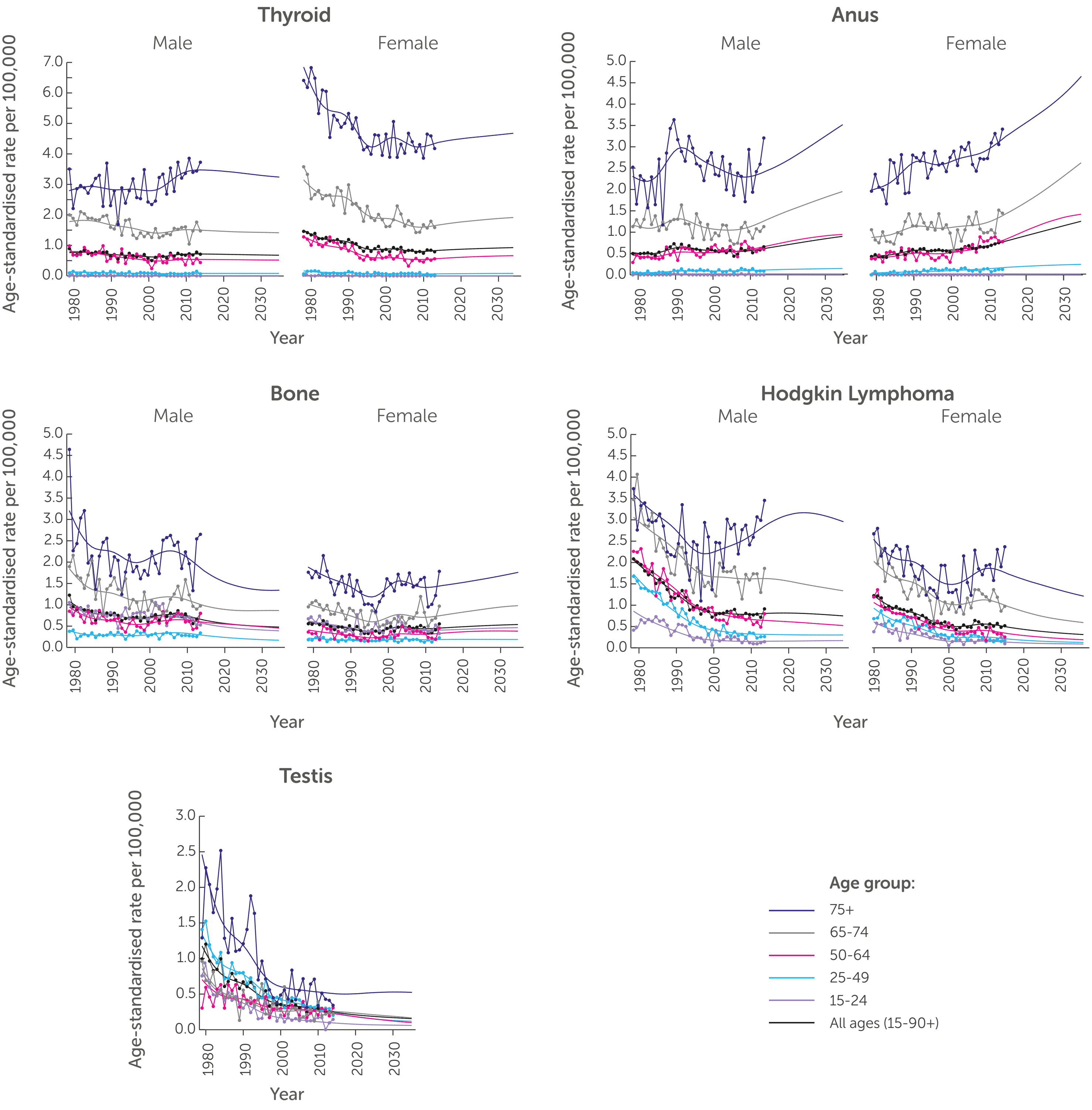

Supplement: Supplementary Material E [file bjc2016304x6.pdf]
