# Supplementary figures and images for: Cancer incidence and mortality projections in the UK until 2035
Source: Br J Cancer. 2016 Oct 11;115(9):1147–55. doi: 10.1038/bjc.2016.304 (PMC5117795; doi:10.1038/bjc.2016.304)

Average registration completeness over an 8 year period by cancer site

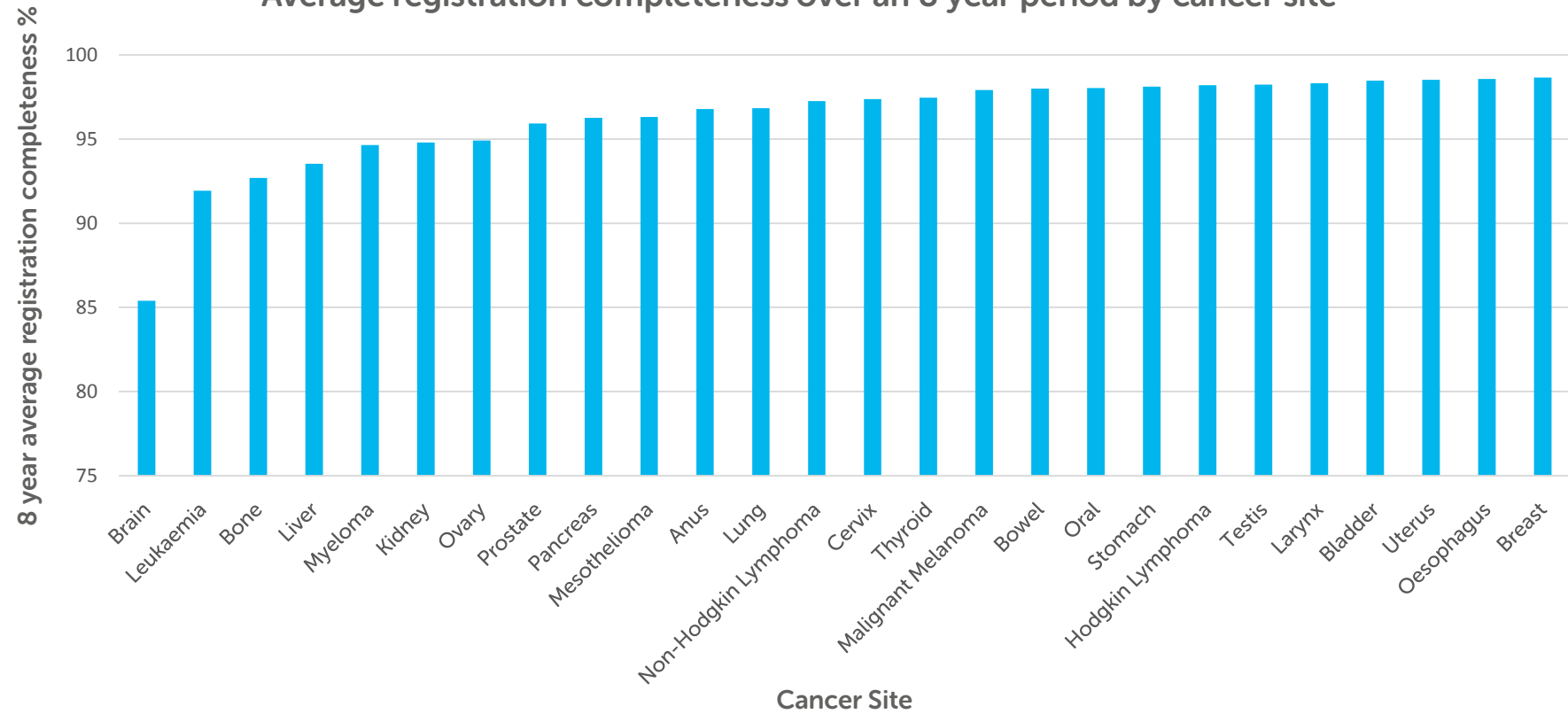

Supplement: Supplementary Material G.2 [file bjc2016304x10.pdf]
