# Supplementary material for: Cancer incidence and mortality projections in the UK until 2035
Source: Br J Cancer. 2016 Oct 11;115(9):1147–55. doi: 10.1038/bjc.2016.304 (PMC5117795; doi:10.1038/bjc.2016.304)
Supplement: Supplementary Materials B, F, G, and H [file bjc2016304x13.docx]

**Supplementary Materials B, F, G, and H**

*Supplementary Material B*

There were several assumptions made throughout the course of the modelling. We explored the impact of these by using a dataset truncated at 1998 to project the 16 years of data up to and including 2014. We assumed that the model parameters which most accurately projected the data over the period 1999-2014 would be the most appropriate choice for projecting the data from 2015 to 2035. Møller et al., (2003) demonstrated that good performance with a method for one time period will generalise to other time periods. As such, this assumption has previously been shown to be valid.

The modelling assumptions which we tested whether they were better able to project data from 1999 to 2014:

- log or a power 5 link function
- levels of attenuation of the trend (2%, 4%, 6%, 8%, 10%, 12%, 14%, and 16%)
- the number of knots in the spline for each age (7, 6, or 5), period (5 or 4) and cohort (3 or 2) effect.

With the power 5 link function, the fitted means may be non-zero, which will have the effect of making the model ill-posed, and therefore it can fail to converge (Sasieni, 2012). We have multiplied the regularisation parameter by a small background constant to enable the model to converge (see Supplementary Material A). The default parameters in the apcspline command are 6, 5, 3 (for age, period, and cohort, respectively), as this was believed to provide the best trade-off between model flexibility and over-fitting (Sasieni, 2012).

We first optimised for the link functions and the trend attenuation, followed by the number of knots in the spline (see Supplementary Material B.1 for incidence, Supplementary Material B.2 for mortality).

For each cancer, we summed the absolute error in the number of cases across years. We favoured this absolute measure above a relative measure, in order to avoid more rare cancers unduly influencing the model choice. When testing between the incidence data, breast and prostate data were not included, as these were both fit with a log model owing to the failure of the power model to converge (without using a large background constant) due to the modified datasets used to account for the effects of screening. The default attenuation and numbers of knots were used for the incidence projections for these sites.

We then sought the model with the smallest deviation between the number of projected cancers and the actual number of cancers recorded over the period.

Supplementary Material B.1 demonstrates the log link function, with 10% dampening, and 7, 5, and 3 knots for age, period and cohort was the most accurate for projecting incidence data 1999-2014. For the mortality data, Supplementary Material B.2 demonstrates that the log link function, with 6% dampening, and 6, 5, and 3 knots for age, period and cohort was more accurate overall in projecting mortality data from 1999-2014. We used these parameters to project the incidence and mortality data, respectively.

*Supplementary Material F*

We calculated age standardised rates per 100,000 15-90+ year olds for incidence and mortality using both the European Standard Population (ESP) 1976 and the ESP 2013 to understand the impact of using these different standard populations. We additionally show the ratio between these rates. Supplementary Material F.1 shows this for incidence data, and Supplementary Material F.2 shows the same for mortality data.

Apart from testis and cervix cancer incidence, the ESP 1976 incidence and mortality ASRs are consistently lower than the ESP 2013 ones. Testis and cervix cancers predominantly affect younger people. In comparison to the ESP 1976, the ESP 2013 weights older age groups more heavily. This is to reflect that a higher proportion of our population now fall into these older age groups in comparison to 40 years ago.

*Supplementary Material G*

Incidence datasets have year-on-year changes due to late registrations. A late registration is when a cancer case is added to a data set from a previous year. Late registrations can occur for several reasons including administrative delay, or the cancer biology not mapping clearly onto the clinical coding, which means it is time-consuming to complete the registration process accurately. This is particularly problematic for haematological malignancies. Late registration can create an artificial downward trend for the most recent years. As the age-period-cohort model is sensitive to the most recent trend in the data (as opposed to the average trend across the entire period of observed data), this can result in projected decreases in incidence or mortality rates. This issue was highlighted by Oliver et al., (2013), when they repeated the projections of Mistry et al., (2011) using a more recent (and therefore more complete) English dataset. With this they were unable to replicate the downwards trends in haematological malignancies, instead reporting increases in all of these malignancies (Oliver et al., 2013).

Here we quantified late registrations using cancer registration datasets for the years 2000-2007 (provided 2009), 2000-2008 (provided 2010), 2000-2009 (provided 2011), 2000-2010 (provided 2012), 2000-2011 (provided 2013), 2000-2012 (provided 2014), 2000-2013 (provided 2015) and 2000-2014 (provided 2016). Datasets were truncated at 2000 as differences prior to this may be driven by changes in disease classification, rather than late registration. For all the datasets where there were overlapping years we calculated the absolute difference between the number of cancers registered in each year.

Inspection of Supplementary Material G.1 reveals that the extent of late registrations is decreasing over time. As such, we anticipate that this late registration problem is less of a problem for the current dataset, compared to the dataset used by Mistry et al., (2011). There are large discrepancies between 2012 and 2013, however, this is likely to be due to under-reporting in 2012 as different cancer registries migrated to a single system (UKIACR, 2015). Importantly, datasets are not complete even many years after their release. This means using a dataset which is two or three years old will reduce, rather than eliminate this problem.

Some cancer types may have more late registrations than others. This could have a particularly distorting effect for low incidence cancers. To explore this further, we calculated an average registration completeness percentage, based on data from 2007 onwards to reflect most recent changes. The difference in cases calculated between each pair of subsequent registration years was divided by the latter of these registration years, and an average was taken of the absolute of all these measures. We used an absolute value as some years registered fewer cases than the previous year. We excluded the ‘other’ cancer category from this analysis, as it is more likely these cancers will be re-classified over time.

Supplementary Material G.2 reveals that brain and leukaemia have the lowest registration completeness. It is possible brain cancer has low registration completeness owing to the difficulties in taking a biopsy from tumours in certain sites, which may cause delays in the registration process. As previously noted, clinical descriptions of leukaemia do not map onto disease categories in a straightforward manner, which may introduce delays in the registration process. As haematological malignancies tend to have relatively low incidence, it is possible that this incomplete registration could lead to falsely decreasing projections, or projections which underestimate the extent of the increases. Non-Hodgkin lymphoma and Hodgkin lymphoma are in the 13th and 20th position respectively, which suggests these cancers are less problematic to register in comparison to leukaemia. Late registration may also be a problem for bone cancer, which is the 3rd least complete and also a low incidence cancer. Liver cancer is the 4th least complete cancer. This low incidence cancer is projected to be one of the fastest accelerating cancers, however, it is possible if there is an under-reporting of liver cancer, the projections under-estimate the extent of the problem.

Ahmad et al., (2013) estimate a 10% change leukaemia registrations and an 8% change in myeloma registrations for 2004 data between 2006 and 2011. However, inspection of Supplementary Material G.1 demonstrates a general trend of the extent of late registration declining over time. As previously noted the large discrepancies between 2012 and 2013 are likely to be a result of the migration to a single cancer registration system in England. Improvements in the cancer registration process mean that we can now expect a more complete dataset sooner. These improvements are already beginning to show in the reduced discrepancies between 2013 and 2014.

Basing projections on earlier registration years is likely to be a sub-optimal solution; Supplementary Material G.1 demonstrates that the problem of late registrations persists for several years (albeit to a lesser extent). As there is already an approximate lag in cancer registration of approximately 18 months, any published figures based on historical datasets will inevitably be out of date.

Caution is warranted when interpreting trends in projections owing to the late registration problem, and projections should be monitored and updated regularly to improve accuracy. However, given the recent improvements in cancer registration processes, it is unlikely that the late registration problem invalidates projections, even for rare cancers.

*Supplementary Material H*

Projection intervals were generated by first calculating the differences between the observed ASRs for 1999-2014 and the ASRs projected for 1999-2014 using 1979-1998 observed data only. Linear regression was then used to determine the trend over time in these differences and to predict what the differences between the projected data and the observed data would be for 2015-2035. The projected differences were then added to and subtracted from the ASRs that were projected using the full 1979-2014 data to create a lower and upper bound. These projection intervals are only intended as crude estimators of uncertainty and are not comparable to confidence intervals.

Supplementary Materials H.1 and H.2 show these projection intervals by site for incidence and mortality ASRs, respectively.
